# A multiparametric extraction method for Vn96-isolated plasma extracellular vesicles and cell-free DNA that enables multi-omic profiling

**DOI:** 10.1038/s41598-021-87526-y

**Published:** 2021-04-13

**Authors:** Jeremy W. Roy, Catherine A. Taylor, Annie P. Beauregard, Surendar R. Dhadi, D. Craig Ayre, Sheena Fry, Simi Chacko, Gabriel Wajnberg, Andrew P. Joy, Ngoc-Nu Mai-Thi, Nicolas Crapoulet, David A. Barnett, Anirban Ghosh, Stephen M. Lewis, Rodney J. Ouellette

**Affiliations:** 1 Atlantic Cancer Research Institute, 35 Providence St., Moncton, NB E1C 8X3 Canada; 2 Department of Chemistry and Biochemistry, Université de Moncton, Moncton, NB Canada; 3 Beatrice Hunter Cancer Research Institute, Halifax, NS Canada; 4 Present Address: Fisheries and Oceans Canada, Aquatic Animal Health, Moncton, NB Canada; 5 Present Address: Immunology, Genetics and Molecular Sciences, University of Medicine and Health Sciences, Basseterre, St. Kitts and Nevis; 6 Present Address: Specialized Health Services Directorate, Health Canada, Ottawa, ON Canada

**Keywords:** Bioinformatics, Isolation, separation and purification, Mass spectrometry, Proteomic analysis, Sequencing, Biological techniques, Biomarkers, Oncology

## Abstract

Extracellular vesicles (EVs) have been recognized as a rich material for the analysis of DNA, RNA, and protein biomarkers. A remaining challenge for the deployment of EV-based diagnostic and prognostic assays in liquid biopsy testing is the development of an EV isolation method that is amenable to a clinical diagnostic lab setting and is compatible with multiple types of biomarker analyses. We have previously designed a synthetic peptide, known as Vn96 (ME kit), which efficiently isolates EVs from multiple biofluids in a short timeframe without the use of specialized lab equipment. Moreover, it has recently been shown that Vn96 also facilitates the co-isolation of cell-free DNA (cfDNA) along with EVs. Herein we describe an optimized method for Vn96 affinity-based EV and cfDNA isolation from plasma samples and have developed a multiparametric extraction protocol for the sequential isolation of DNA, RNA, and protein from the same plasma EV and cfDNA sample. We are able to isolate sufficient material by the multiparametric extraction protocol for use in downstream analyses, including ddPCR (DNA) and ‘omic profiling by both small RNA sequencing (RNA) and mass spectrometry (protein), from a minimum volume (4 mL) of plasma. This multiparametric extraction protocol should improve the ability to analyse multiple biomarker materials (DNA, RNA and protein) from the same limited starting material, which may improve the sensitivity and specificity of liquid biopsy tests that exploit EV-based and cfDNA biomarkers for disease detection and monitoring.

## Introduction

Biomarkers have been applied to the diagnosis and management of a wide variety of diseases, including cancer, cardiovascular disease, neurodegenerative diseases, immunological diseases and many others. Currently biomarkers for diseases such as cancer are detected by utilizing tissue biopsies; however, tissue biopsies are invasive and suffer from limitations such as sampling bias, failure to capture tumour heterogeneity, inability to perform screening or early detection, cost, and the inability to monitor changes as the disease progresses once the primary tumor has been removed or treated^1^. Furthermore, tissue biopsy comes with inherent drawbacks and risks to the patient, such as pain, the potential for complications, or the need for anesthesia^2^. In many instances, tissue biopsy is not feasible due to inaccessibility of the tumor or due to the patient’s inability to withstand the procedure.

Liquid biopsy refers to the collection of a biofluid and the subsequent analysis of disease biomarkers that are present in the biofluid for disease detection and/or monitoring. In contrast to tissue biopsy, liquid biopsies are minimally-invasive, proposed to allow a comprehensive sampling of tumour heterogeneity, more readily permit longitudinal sampling, are cost-effective, and require only moderate training^3,4^. Liquid biopsy typically makes use of blood samples; however, other biofluids can be used, such as urine, saliva, breast milk, and cerebrospinal fluid. Certain types of liquid biopsies (such as urine) typically yield substantial volumes of fluid, whereas others (such as blood and cerebrospinal fluid) may only provide a few milliliters of biofluid for downstream analyses. Similarly, biobanks that collect and curate patient biofluids, such as plasma, are frequently only able to provide a limited volume of sample to researchers in order to maximize the number of researchers and research projects that can access the samples. Therefore, it is important to devise liquid biopsy protocols that extract the most information from a limited sample volume.

Beyond the collection and analysis methods employed, evaluating clinically-relevant biomarkers relies on the comprehensive analysis of information obtained from the molecular profiling of DNA, RNA and protein. Several approaches are currently being used to isolate material from biofluids for liquid biopsy testing, including the capture and analysis of cell-free DNA (cfDNA), circulating tumour cells, and extracellular vesicles (EVs). A drawback of using cfDNA for a liquid biopsy test is that only DNA mutations can be analyzed. In contrast, circulating tumour cells provide material for DNA, RNA, and protein analyses, but their limited numbers and difficult isolation^5,6^ are hurdles for widespread use in liquid biopsy testing. Since circulating EVs contain DNA, RNA, and protein derived from the site of disease^7–10^ and since EVs are often present in high abundance in the biofluids of affected patients^11^, EVs represent an excellent starting material for liquid biopsy-based molecular testing. Moreover, the ability to assay multiple biomarker materials (i.e. a multiparametric extraction of DNA, RNA, and/or protein and subsequent multi-omic analyses) from the same sample should provide a comprehensive analysis of the molecular profile of a particular patient’s disease. To our knowledge several articles have discussed the potential use of a multi-omic approach to discover novel circulating biomarkers^12,13^; however, there is a lack of reports demonstrating the development of protocols that combine EV isolation with multiparametric extraction methods to enable this multi-omic approach.

Herein we describe a multiparametric extraction protocol that can isolate DNA, RNA and protein from EVs and cfDNA isolated from the same starting material: in this case, human plasma. Our approach for the isolation of biomarker-containing material from plasma for multiparametric extraction and analyses relies on the Vn96 synthetic peptide, which has been shown to isolate both EVs^14^ and cfDNA^15^ from biofluids. We show that we can perform diagnostically-relevant tests and multi-omic profiling on the extracted material, including droplet digital PCR (ddPCR) for DNA, small-RNA sequencing for analysis of miRNA, and mass spectrometry for characterization of EV-associated proteins.

## Methods

### Plasma samples

Human plasma derived from whole blood collected in EDTA-tubes was purchased from Innovative Research Inc. (Novi, MI, USA). Whole blood was processed within 1 h of collection by centrifugation at 5000× g for 15 min at 4 °C in order to separate the plasma fraction.

All methods were carried out in accordance with the Canadian Research Tri-Council policy on ethical conduct for research involving humans (https://ethics.gc.ca/eng/policy-politique_tcps2-eptc2_2018.html). Approval for this study was obtained from the Research Ethics Board of Vitalité Health Network and written informed consent was obtained from the patients. Donors were not fasted. See Table 1 for donor information.

**Table 1:** Donor plasma.

Donor	Gender	Age	Ethnicity
a	F	32	Hispanic
b	M	48	Caucasian
c	M	27	Black
d	F	24	Hispanic

### Ultracentrifugation (UCF) and sucrose-cushion UCF (scUCF)

Plasma (1 mL) was diluted to ~ 10 mL with 0.1 µm filtered 1 × PBS and either directly transferred to an ultracentrifuge tube (UCF) or overlayed on top of 0.5 mL 30% sucrose solution (scUCF; ρ = 1.127) and subjected to ultracentrifugation at 100,000 × g for 2 h at 4 °C using a Beckman Coulter Ultracentrifuge equipped with a SW40Ti rotor (k-factor = 277.5). The UCF EV pellet was washed once with PBS followed by a second ultracentrifugation at 100,000 × g for 2 h and the resulting pellet resuspended in 2 M NaCl overnight at 4 °C for nanoparticle tracking analysis (NTA), or protein lysis buffer [0.125 mM Tris–HCl; pH 6.8, 2% SDS, 1 × Protease Inhibitor Cocktail Set III (PICIII)] for Western blot analyses. For scUCF, the sucrose layer was collected (~ 1 mL) and diluted 1:10 with PBS and subjected to ultracentrifugation at 100,000 × g for 2 h and the resulting pellet solubilized in 2 M NaCl overnight at 4 °C for nanoparticle tracking analysis (NTA), or protein lysis buffer for Western blot analyses.

### Vn96-mediated EV isolation and characterization

**Figure 1:**
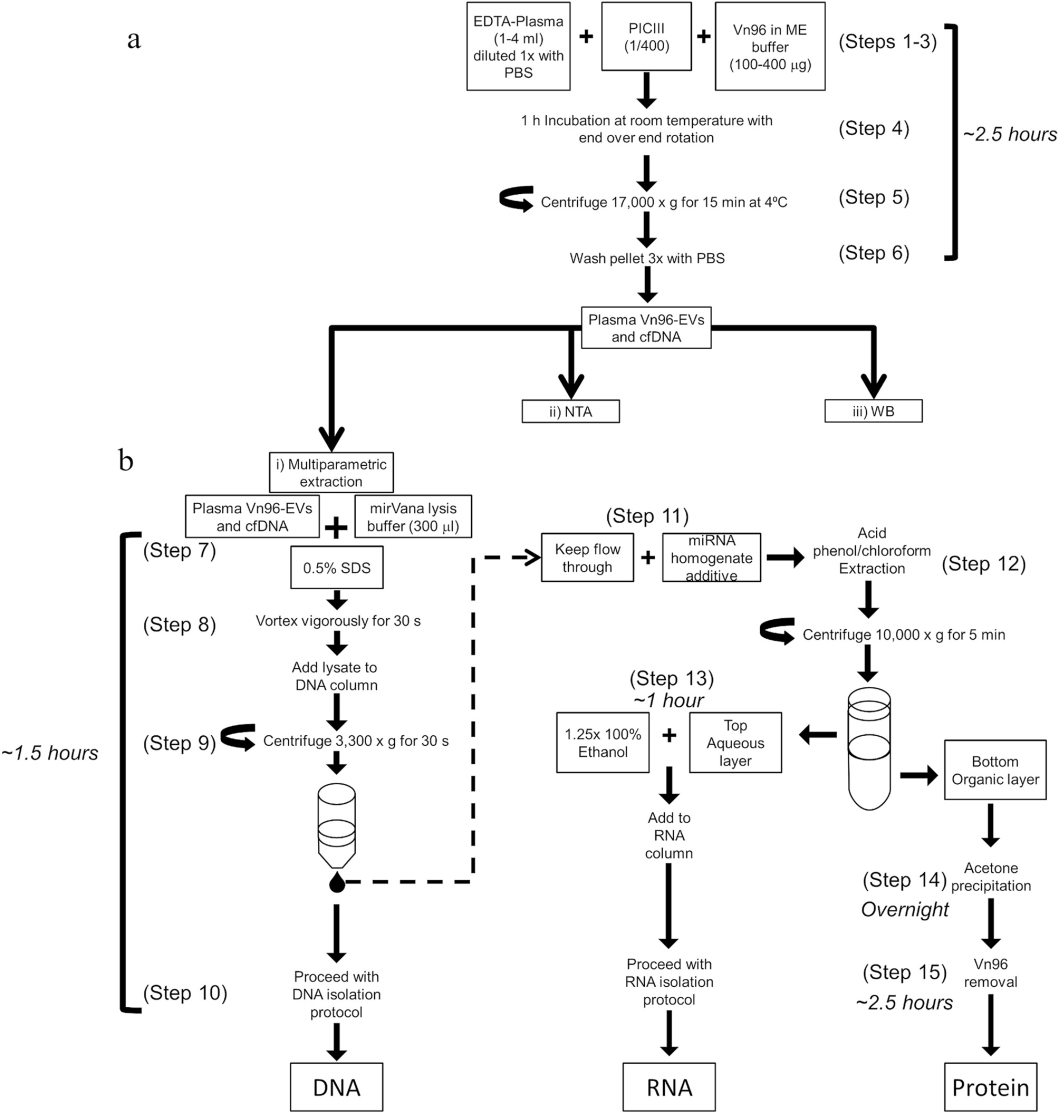
Schematic representation of the simplified protocol for the use of (**a**) Vn96 in plasma and (**b**) subsequent multiparametric extraction of DNA, RNA and Protein.

EVs were extracted using the ME kit for EV isolation (New England Peptide, Gardner, MA, USA), which uses the synthetic peptide Vn96 for an affinity capture method^14^ for the isolation of EVs, EV-associated nucleic acid, and EV proteins from a variety of biofluids. Figure 1a presents our simplified protocol used for isolating EVs from plasma collected in EDTA tubes. Plasma (1–4 mL) from each donor was diluted 1:1 with RNAase-free PBS (1 ×) pre-filtered at 0.1 µm (Fig. 1a, Step 1). Protease Inhibitor Cocktail Set III (1:400; MilliporeSigma) was added to each diluted donor plasma (Fig. 1a, Step 2). Vn96 (100 µg per mL of undiluted plasma), which was suspended at a stock concentration of 2.5 mg/mL in ME buffer, was added to the samples (Fig. 1a, Step 3) and incubated for 1 h with end-over-end rotation at room temperature (Fig. 1a, Step 4). Samples were centrifuged at 17,000 × g (k-factor = 743) for 15 min at 4 °C (Fig. 1a, Step 5). Supernatant was discarded and Vn96-EV pellets were washed 3 times with 2 mL of 1 × PBS pre-filtered at 0.1 µm followed by centrifugation at 17,000 × g for 10 min at 4 °C (Fig. 1a, Step 6). The resulting Vn96-EV and cfDNA pellet was solubilized in either (1) miRVana lysis buffer for single-parametric RNA extraction (protocol provided in miRVana miRNA extraction kit) or multiparametric extraction (see below), (2) 2 M NaCl and incubated overnight at 4 °C for nanoparticle tracking analysis (NTA), or (3) protein lysis buffer for Western blot analyses. A scrambled peptide (Scr; New England Peptide) and ME buffer (without peptide) were included as negative controls.

### Multiparametric extraction

Disruption and lysis of the Vn96-EV and cfDNA pellet (from Fig. 1a, Step 6i) for multiparametric (MP) extraction was performed at room temperature in 300 µL miRVana lysis buffer (Fig. 1b, Step 7) in the presence of 0.5% RNAase-free SDS, while vortexing vigorously for 30 s making sure the pellet was completely dissolved (Fig. 1b, Step 9). The resulting lysate was then added to a DNA-specific column designed to capture small fragments of circulating DNA. In this case, we chose DNA columns from the Plasma/Serum Cell-Free Circulating DNA purification kit (Norgen Biotek). After centrifugation at 3300× g for 30 s the flow-through from the DNA column was collected for recovery of RNA and protein, whereas the Vn96-EV DNA retained on the column was further processed following the manufacturer’s protocol (Fig. 1b, Step 10). The flow-through from the DNA column was then used to isolate RNA and protein. RNA was isolated following the acid phenol/chloroform extraction protocol described for Total RNA in the miRVana kit (Fig. 1b, Steps 11–13). The organic fraction from the acid phenol/chloroform extraction was used to extract protein using an acetone precipitation method (^16^; Fig. 1b, Step 14). Briefly, the organic fraction was stored at − 20 °C in 2.5 times the volume of ice-cold acetone overnight to precipitate proteins, followed by centrifugation for 10 min at 14,000 × g and the supernatant was discarded. Protein pellets were washed once in ice-cold acetone and solubilized in a solution containing 8 M Urea, 1% SDS, and 100 mM Tris–HCl (pH 8) at a ratio of 50 µL per mL of starting plasma and then brief sonication at 50 °C. Since the added Vn96 peptide would contribute to the total amount of captured protein (data not shown) we depleted the Vn96 peptide from the isolated protein samples by passing the sample through a 3 kDa cut-off filter (MilliporeSigma) and buffer exchanged with 100 mM ammonium bicarbonate four times (Fig. 1b, Step 15).

### Quantification of DNA, RNA and protein

The amount of DNA isolated per mL of plasma was determined using the Taqman Copy Number RNase P Detection kit (ThermoFisher Scientific) adapted for use with ddPCR and using a standard curve prepared from human genomic DNA to extrapolate sample DNA concentration. The amount of RNA isolated per mL of plasma was determined using Qubit microRNA assay kit (ThermoFisher Scientific). The resulting protein concentration was determined using a BCA Protein assay (Fisher Scientific).

### Nanoparticle tracking analysis (NTA)

The size distribution and concentration of EVs purified from plasma by UCF, scUCF, Vn96, ME buffer and Scr peptide (from Fig. 1a, Step 6ii) were measured using a NanoSight NS300 equipped with a 405 nm laser (Malvern Panalytical Ltd.,United Kingdom) and analyzed using Nanosight software (v3.2, https://www.malvernpanalytical.com/en/support/product-support/software/NanoSight-NTA-software-update-v3-2, accessed on 28/01/2021). Samples were prepared for NTA analysis by diluting the stock material in particle-free water until the concentration was between 1 × 10^8^ and 1 × 10^9^ particles/mL and six videos of 30 s were captured, analyzed with Gain = 512 and shutter at 1300, and averaged. At least two different dilutions resulting in good correlation of the stock concentration (% coefficient of variance (%CV) < 25%) were included in the analysis for each sample. Size distribution data was analyzed by normalizing the concentration of particles of different diameters with bin widths of 1 nm to the highest concentration observed within a 1 nm bin and then taking the average of each measurement. The average of each technical replicate (n = 2) was averaged with each biological replicate (n = 3) and expressed as particles per mL of plasma ± standard error of the mean (SEM).

### Western blotting

Protein lysates obtained from EVs isolated by UCF, scUCF, Vn96, ME buffer and Scr peptide (from Fig. 1a, Step 6ii) were separated by sodium dodecylsulfate polyacrylamide gel electrophoresis (SDS-PAGE) and analyzed by Western blotting using the BioRad mini-gel system for canonical EV protein markers as well as calnexin, an endoplasmic reticulum protein that is not localized in EVs. All antibodies were supplied by Santa Cruz Biotechnology (Dallas, TX) except for antibodies to Flotillin-1 (Cell Signaling Technologies; cat # 18634S) and Calnexin (Abcam, cat # 22,595). Santa Cruz antibodies included CD63 (sc-5275), CD9 (sc-59140), and HSC70 (sc-7298). All primary antibodies were incubated overnight at 4 °C in 5% milk/PBS at a dilution of 1:1,000, with the exception of HSC70 which was used at a dilution of 1:500. Goat anti-rabbit and goat anti-mouse secondary antibodies were purchased from Jackson ImmunoResearch Inc. and used at 1:10,000. Clarity Western ECL blotting substrate (Bio-Rad, Hercules, CA) was used for Western blot detection.

### Transmission electron microscopy

Transmission electron microscopy (TEM) for the characterization of EVs isolated from plasma was performed according to Ghosh et al*.* (2014). EVs were eluted from Vn96-EV pellets by the addition of 100 µL of 2 M NaCl, followed by vigorous vortexing for 1 min and incubation on a shaker plate at room temperature for 1 h. The tube was centrifuged at 17,000 × g for 15 min and the supernatant containing the EVs was collected. Proteinase K (100 µg; Life Technologies) treatment was performed by adding 5 µL and incubating the tubes at 50 °C for 2 h and 24 h to remove residual Vn96. NaCl and Proteinase K were removed by buffer exchange using 3 kDa spin-filters (Amicon Ultracel columns from Millipore). The desalted EVs (in water) were deposited onto formvar/silicone monoxide-coated 200 mesh copper grids (Electron microscopy Sciences) for 10 min, followed by 2–3 washes with water. EVs were fixed with 3.7% formalin followed by 2 washes with water. The samples were contrasted with 2% Uranyl Acetate (w/v) to visualize. UCF EVs were suspended in water and deposited onto grids as described above. All solutions were 0.1 µm filtered to avoid particulate deposits on the grids. The dried grids were viewed using JEOL 6400 electron microscope.

### ddPCR: KRAS, EGFR, TP53

EV DNA (5.5 µL) was used to detect *KRAS*, *EGFR* and *TP53* wildtype (WT) genes using ddPCR. A probe for *KRAS* WT was developed in-house using lock-nucleic acid technology (Forward Primer: 5′-AACCTTATGTGTGACATGTTCT-3′; Reverse Primer: 5′-GATTCTGAATTAGCTGTATCGTCAAG-3′; Probe: HEX-5′-AGCT + G + G + TG + GC-3′-IABkFQ). *EGFR* WT (assay Id: dHsaCP2000020) and TP53 WT (assay Id: dHsaCP2506903) assays were purchased from Bio-Rad. Resulting amplicon copies were normalized to the amount of DNA (ng) input for each reaction.

### Next-generation small RNA sequencing (sRNAseq)

RNA quality was assayed on Agilent Tape Station 2200 using RNA HS screen tape assay. RNA (25 ng) was used to prepare a small RNA library using Clean Tag Small RNA library kit (TriLink biotech) following manufacturer recommended conditions. Adapter ligated RNA was reverse transcribed using Ion Torrent specific primer. cDNA was then amplified using Ion convert barcode primers containing a unique index to allow libraries to be pooled during Ion Torrent sequencing (Life Technologies). Library quality control (QC) was assayed on Agilent Tape Station using D1000 screen tape. Equally-pooled libraries (pmol) were clonally amplified onto Ion Sphere Particles (ISPs) supplied by Ion Pi HiQ OT2 kit. ISPs were then loaded onto Ion PI chip and sequenced using Ion Torrent Proton Sequencer.

### Bioinformatic analysis of sRNAseq

Raw reads from sRNAseq were processed with the Torrent Suite Software (v5.4.0, https://www.thermofisher.com/ca/en/home/life-science/sequencing/next-generation-sequencing/ion-torrent-next-generation-sequencing-workflow/ion-torrent-next-generation-sequencing-data-analysis-workflow/ion-torrent-suite-software.html, accessed on 28/01/2021), which runs the Torrent Mapping Alignment Program (TMAP) using the human reference genome version GRCh37/hg19. The RNASeqAnalysis plugin from the Torrent Suite Software (v5.4.0.2) was subsequently used to map the data with human miRBase 20^17^ and generate read counts for each miRNA. Removal of adapter sequences and triage of reads 15 to 30 bp in length were also performed using Cutadapt (v1.8.1)^18^ and Picard tools (release 1.113)^19^. Mapping was conducted with Bowtie 2 (v2.2.5)^20^ and read counts were obtained with featureCounts (v1.4.6-p4)^21^. R statistical environment (v3.4.1) was used to calculate the variance between the normalized read counts of miRNA with trimmed mean of M-values (TMM) normalization method^22^ of Bioconductor package edgeR (v3.18.1)^23^. The sRNAseq data have been deposited in the NCBI Gene Expression Omnibus (GEO) under the accession number GSE133991 (https://www.ncbi.nlm.nih.gov/geo/query/acc.cgi?acc=GSE133991).

### Mass spectrometry (MS) analysis

The protein fraction (Fig. 1b, Step 15) was prepared for SDS-PAGE fractionation by re-solubilizing samples in SDS-PAGE loading buffer [2% SDS (w/v), 50 mM Tris–HCl, 0.1% bromophenol blue (w/v), 10% glycerol (v/v) and 100 mM dithiothreitol (DTT) (w/v)]. Samples (25 µg) were processed on 4–20% pre-cast electrophoresis gels (Cedarlane, Burlington, ON), run for 2 h at 100 V, fixed with 50% methanol containing 5% acetic acid, stained with EZ-blue gel staining reagent (Sigma-Aldrich, Oakville, ON) and de-stained overnight in deionized water. Each gel lane was excised into twelve equal sized bands which were then treated with 10 mM DTT for reduction of disulfide bonds, followed by alkylation of cysteine residues with 25 mM iodoacetic acid. Sequencing-grade trypsin (Promega, Madison, WI) was added (1 ug) to each gel band to carry out protein digestion at 37 °C for 16 h. Following tryptic digestion, the supernatant was collected and remaining gel-bound peptides were extracted three times using 50 µL aliquots of 50% acetonitrile containing 5% acetic acid and pooled with the initial collection. The combined extract was then concentrated by vacuum centrifuge and further processed using mini-spin filter cartridges (Canadian Life Sciences, Mississauga, ON) containing C-18 resin for clean-up by solid-phase extraction. Upon completion of the sample loading and wash steps on the C18 cartridge, peptides were eluted with 70% acetonitrile and concentrated again in a vacuum centrifuge. The samples were then reconstituted to a volume of 50 µL containing 1% aqueous acetic acid and stored at − 80 °C. Analyses of tryptic digests for bottom-up protein identification were performed by nanoLC-MS/MS using a Proxeon Easy Nano-LC II interfaced to a Quadrupole Orbitrap (Q-Exactive, Thermo-Fisher Scientific, San Jose, CA) mass spectrometer. A volume of 5 µL of each sample was injected onto a narrow bore (100 µm inner diameter (i.d.) × 20 mm long) C-18 pre-column packed with 5 µm Reprosil-Pur resin (Thermo-Fisher Scientific, San Jose, CA). Chromatographic separation was achieved on a Thermo-Scientific Easy C18 analytical column with dimensions of 75 µm i.d. and 100 mm long. The gradient LC elution began with 95% solvent A (0.1% aqueous formic acid) 5% solvent B (0.1% formic acid, 10% water and 90% acetonitrile) which was increased linearly to 35% solvent B over 60 min followed by 90% solvent B for 10 min and re-equilibrated to 95% solvent A for 10 min. The LC flow rate was held at a constant value of 300 nL/min. All solvents were of LC–MS grade from VWR (Mississauga, ON). The 15 µm i.d. electrospray (ESI) emitter of the Q-Exactive was biased between 1.6 and 1.9 kV and was positioned approximately 2 mm from the heated (250 °C) ion transfer capillary. The S-lens of the mass spectrometer was maintained at 100 V. The Orbitrap mass analyzer was calibrated in positive ion mode at 70 k resolution every three days using Thermo LTQ-VELOS positive calibration solution (Thermo-Fisher). Mass spectrometric data was acquired in “data dependent acquisition” (DDA) mode whereby a full mass scan from 400 to 1200 Thomsons (Th) was followed by the acquisition of fragmentation spectra of the ten most abundant precursor ions with full-scan intensities greater than a threshold of 60,000. Precursor ion spectra (i.e., full scan) were collected at a resolution of 70 k (@ 200 amu) and a target value of 1E^6^. Peptide fragmentation was performed at 27 eV within the high energy collision induced dissociation (HCD) collision cell. The subsequent MS/MS spectra were collected in the Orbitrap analyzer at a resolution setting of 17.5 k and target value of 1E^5^. The *m/z* values of the selected peptide precursor ions were placed on a dynamic exclusion list for a period of 40 s to maximize the number of fragmentation spectra collected for unique peptides. Each excised band (12) was analyzed by MS in triplicate (MS experimental replicate) for each donor (4) sample to yield a total of 144 raw data files.

Raw data files from the mass spectrometer were searched against the Swissprot Homo sapiens database (version July 2017, 13,081 entries) accessed from ‘The Universal Protein Resource’ (www.uniprot.org). The searches were completed by using Proteome Discoverer 2.0 (Thermo-Fisher Scientific, San Jose, CA) employing the Sequest HT algorithm. Searches were performed using the following settings: 2 allowed missed cleavage sites, 10 ppm precursor mass tolerance and 0.8 Da fragment mass tolerance, dynamic modifications of methionine oxidation (+ 15.99 Da) and N-terminal acetylation (+ 42.01 Da) as well as a static modification of cysteine carboxymethylation (+ 58.005 Da). Proteome discoverer used the Percolator algorithm to calculate a false discovery rate (FDR) of 0.1% based on the results of a decoy (reversed) database search. The common set of proteins from the three MS experimental replicates were prepared for each donor sample and used for analysis. REVIGO was used for gene ontology and a web-based tool (http://bioinformatics.psb.ugent.be/webtools/Venn/, accessed on 28/01/2021) was used to prepare Venn diagrams^24^. All raw data files, search files and database files have been uploaded and made publicly available on the Mass Spectrometry Interactive Virtual Environment (MassIVE, www.massive.ucsd.edu) and assigned a ProteomeXchange ID of PXD015204.

### Statistical analysis

Statistical significance was determined by *t-*test analysis for particle numbers from NTA data. A *p*-value less than 0.05 is considered statistically significant. Single-parametric (SP) and multiparametric (MP) miRNA abundance were compared and analyzed by a linear regression model.

## Results

### Optimization of Vn96-mediated isolation from plasma samples

The utility of the Vn96 synthetic peptide to isolate EVs from numerous biological fluids has been previously demonstrated^14^; moreover, Vn96 has recently been shown to co-isolate cfDNA along with EVs from biofluids^15^. We now describe the development of a multiparametric extraction protocol for use with EVs and cfDNA isolated from healthy donor plasma using the Vn96 peptide.

**Figure 2:**
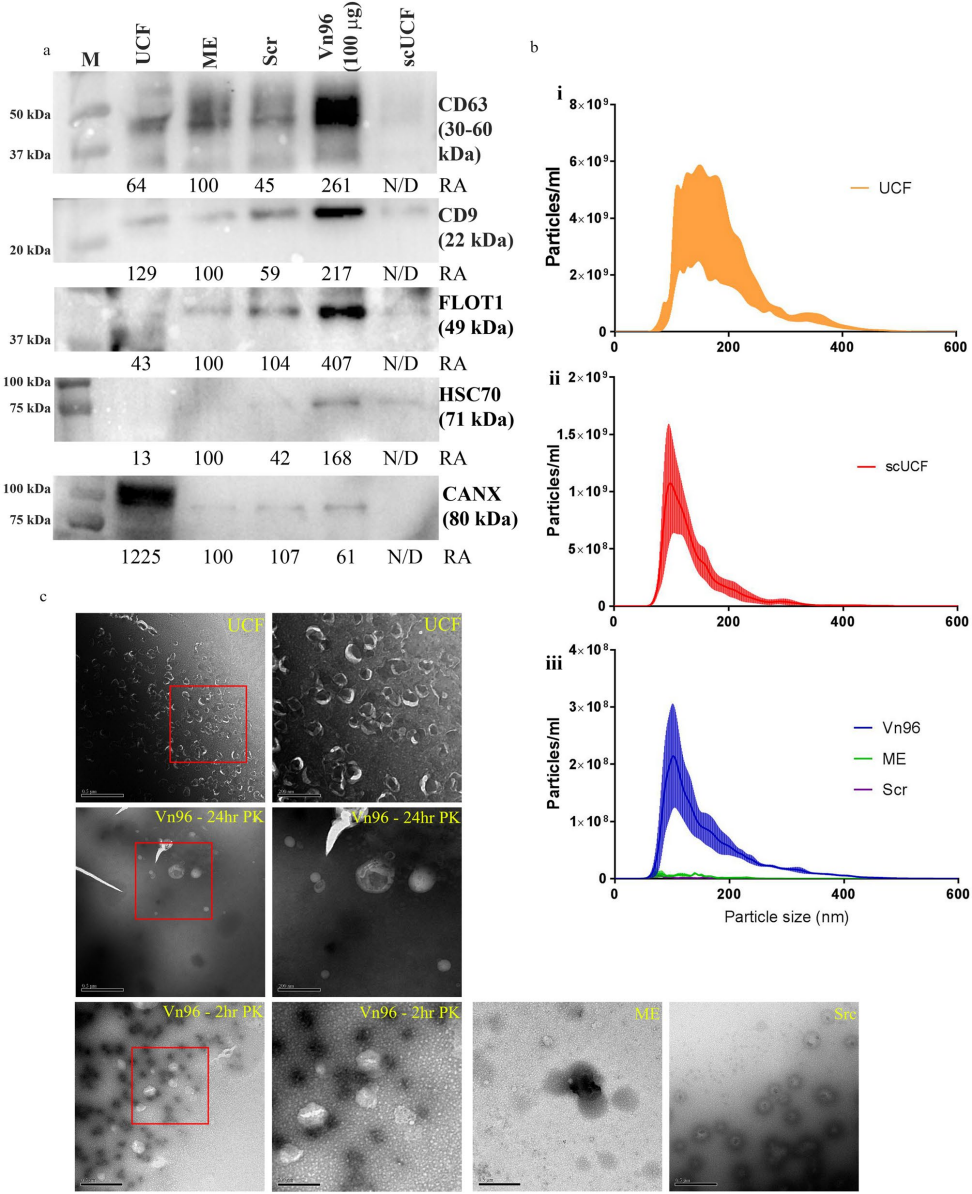
Characterization of plasma EVs isolated by UCF, scUCF and Vn96 methods, all from the same donor plasma (n = 3). (**a**) Representative Western blot comparing different EV isolation methods probing for canonical EV markers: CD63 (65 kDa), CD9 (24 kDa), FLOT1 (50 kDa), HSC70 (73 kDa), and cytoplasmic marker CANX (75 kDa). Mean relative abundance (RA) for three experiments is indicated below each Western blot (ME is set at 100). Full-length blots and all biological replicates are presented in Supplementary Fig. 3. (**b**) NTA results showing the number of particles ± SEM per mL of starting plasma on the y-axis plotted against the size (nm) of particles on the x-axis from UCF (i), scUCF (ii), and Vn96 with appropriate controls (iii). (**c**) Representative TEM images of EVs captured by UCF, Vn96 (2 h and 24 h post proteinase K treatment), ME buffer and Scr peptide.

We first characterized the EVs isolated from plasma using the Vn96 peptide and compared them to EVs isolated using ultracentrifugation methods. Figure 2a shows a representative Western blot (n = 3) in which EVs isolated from healthy human plasma were examined for the presence of canonical EV markers (CD63, CD9, Flotillin1, and HSC70). UCF, Vn96 (100 µg), Scr (scrambled control peptide, 100 µg) and ME (buffer control for peptide-mediated capture) were used to isolate EVs from 1 mL of plasma; 2 mL of plasma was used for scUCF isolation of EVs. The same healthy human plasma was used for each isolation method in order to reduce biological variability and to enable direct comparison of EVs markers. Calnexin (CANX), which is not found in EVs, but in cells and cellular debris^25^, was included as a control for non-EV material. CD63, CD9 and Flotillin-1 (FLOT1) were detected for all EV isolation methods (UCF, Vn96, and scUCF), albeit at a much lower amount in scUCF. The Vn96 synthetic peptide isolated more of these three canonical EV protein markers when compared to UCF, scUCF, Scr, and ME buffer, suggesting a better yield of EVs from plasma using Vn96-based isolation than either UCF or scUCF isolation methods. HSC70 was only significantly detected in Vn96- and scUCF -isolated EVs, consistent with the affinity of Vn96 for heat-shock proteins and heat-shock protein-associated EVs^14^. Surprisingly, CANX was detected in all samples except scUCF, indicating the potential co-isolation of large EVs, apoptotic bodies, or aggregated protein complexes^26^ by the 100,000 × g UCF and the 17,000 × g centrifugation steps required for Vn96-mediated isolation. The presence of co-precipitating protein is likely due to the lack of a plasma pre-clearing step to remove co-sedimenting material prior to EV isolation. Since CD63, CD9, FLOT1 and HSC70 are enriched in Vn96 samples compared to ME and Scr samples, these findings suggest that Vn96 does not merely sediment circulating proteins at 17,000 × g under these conditions, but rather enriches for these EV-specific proteins. Recent publications have also demonstrated co-isolation of lipoproteins (ApoB and ApoA1) with both UCF and Vn96^15,27^, suggesting that the proteomic profile of material isolated by Vn96 may be similar to UCF.

We further characterized the concentration and size distribution (Table 2 and Fig. 2b) of particles isolated by UCF, scUCF and Vn96 using nanoparticle tracking analysis (NTA). There were no statistically significant differences between the isolation methods (UCF, scUCF and Vn96); however, there were statistically significant differences among Vn96 and the ME buffer and Scr controls (*P < 0.05, n = 3). As shown in Fig. 2b there were differences observed in the size distribution of particles for each isolation method. UCF isolated a heterogeneous population of particles, with the majority of particles ranging between ~ 100 and 300 nm, whereas scUCF isolated a more homogeneous population of particles with a peak size of ~ 100 nm. Vn96 isolated a homogeneous population of particles with a peak size of ~ 100 nm that was similar to scUCF, but with a slightly wider size distribution that includes a population of particles slightly larger than those isolated by scUCF.

**Table 2:** NTA using multiple EV capture methods.

Capture method	Number of particles ± SEM (particles/mL of plasma)	Mean size ± SEM (nm)	Mode size ± SEM (nm)
UCF (n = 3)	5.58 ± 2.15 × 10^13^	185 ± 16	151 ± 22
scUCF (n = 3)	7.71 ± 2.14 × 10^12^	138 ± 14	99 ± 5
Vn96 (n = 3)	2.04 ± 0.53 × 10^12^	173 ± 23	108 ± 10
ME (n = 3)	9.01 ± 3.06 × 10^10^*	138 ± 12	108 ± 16
Scr (n = 3)	3.69 ± 1.45 × 10^10^*	124 ± 7	82 ± 6

*P < 0.05.

Lastly, we performed transmission electron microscopy (TEM) on the EVs captured by UCF, Vn96, ME buffer and Scr peptide. In order to visualize EVs captured by Vn96, isolated EVs were treated with proteinase K for 2 h and 24 h to digest residual Vn96, as we have previously shown that visualizing EVs isolated with Vn96 by TEM is not possible without proteinase K treatment^14^. As shown in Fig. 2c, the typical double membrane structure of EVs is found in the material isolated by UCF and Vn96; however, no discernable EVs were identified in the ME buffer and Scr peptide samples.

Together, these results suggest that Vn96 isolates a population of EVs with a size distribution that reflects the presence of EVs and is enriched for canonical EV protein markers such as CD63, CD9, Flotillin1, and HSC70. We have therefore re-confirmed that the Vn96 peptide efficiently isolates EVs and that it can be reliably used with samples of human plasma, which can be used for downstream liquid biopsy tests. Since the ultracentrifugation methods for EV isolation are not amenable to a clinical setting due to the time and specialized equipment required, we further investigated yields of potential biomarker material for clinical diagnostics that are obtained by Vn96-mediated EV isolation.

### Development of a multiparametric extraction method for Vn96-isolated EVs and cfDNA

The majority of liquid biopsy tests rely on a single parameter; however, the same disease may have tests that assay multiple parameters. For example, liquid biopsy tests for colorectal cancer have been developed for DNA (evaluating Sept9 methylation) and protein (abundance of CEA), and new assays have been suggested that examine small RNA^28–30^. Separately these assays demonstrate good sensitivity and specificity; however, very few of these assays have been combined into a single assay format that analyzes multiple parameters from the same starting material, which could lead to gains in both sensitivity and specificity. In order to facilitate the multiplexing of biomarker analyses for different parameters, we chose to develop and characterize a multiparametric extraction protocol to isolate DNA, RNA and protein from the same liquid biopsy sample processed with the Vn96 synthetic peptide (Fig. 1b). It is important to note that Vn96 is able to capture not only DNA associated with EVs, but also cfDNA^15^. We confirmed this property of Vn96 as we are able to recover non-vesicular DNA from EV-depleted plasma that had been supplemented with a synthetic gene fragment (Figure S1); therefore, Vn96 can effectively capture all DNA from a biofluid sample, rendering the Vn96 isolation method more applicable for biomarker analyses beyond a straight-forward EV isolation method. For this reason we have chosen to pursue our multiparametric extraction protocol development for liquid biopsy by employing the Vn96 peptide for EV and cfDNA isolation.

### Quantification of EV cargoes obtained using the multiparametric extraction method

**Figure 3:**
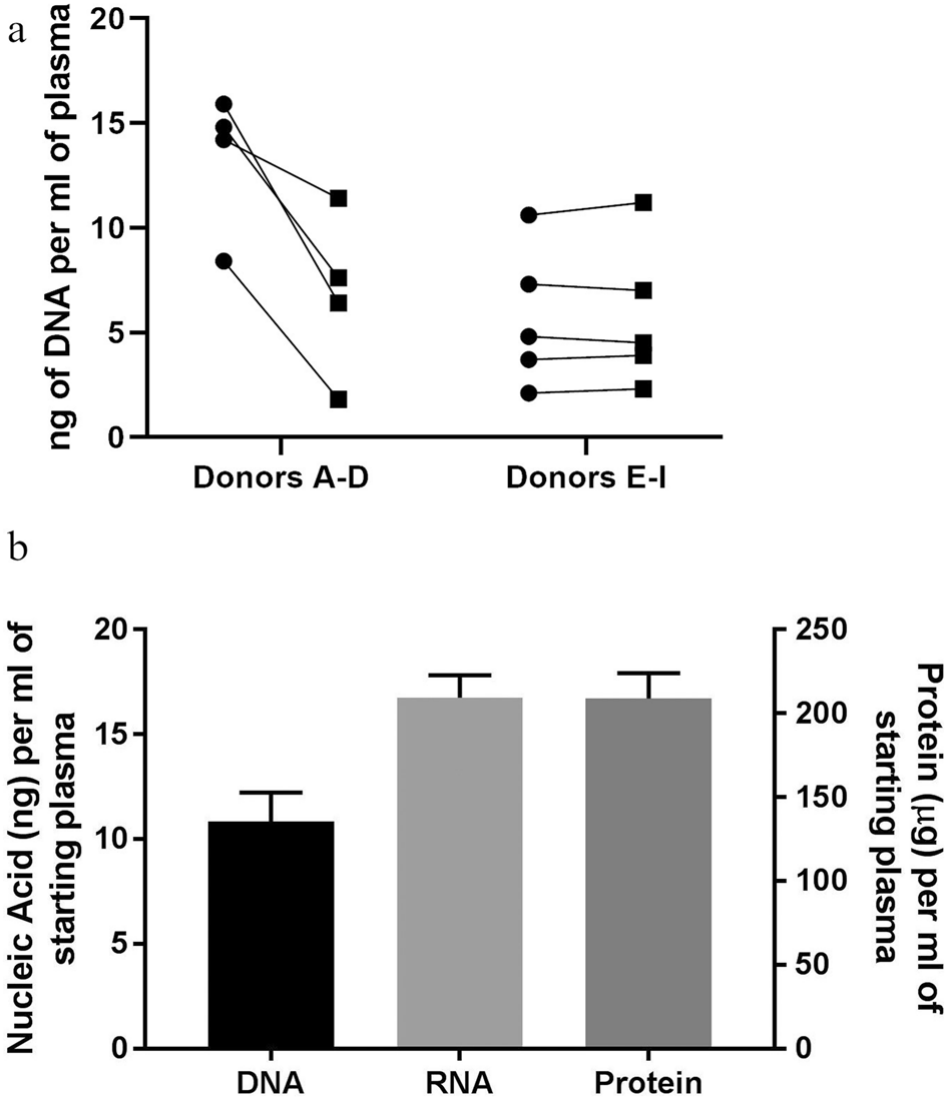
(**a**) Plasma (1 mL) from 9 healthy donors was subjected to centrifugation at 16, 000 × g for 10 min (squares) or not (circles) prior to the addition of Vn96 (100 µg) for EV isolation. DNA was extracted using the Qiagen Circulating Nucleic Acid Kit (Qiagen, cat # 55,114) and quantified with ddPCR using an RNASE P TaqMan Assay. (**b**) DNA, RNA and protein yields per mL of starting plasma. Yields of DNA and RNA (ng) on the left y-axis and protein (µg) on the right y-axis ± SEM per mL of starting plasma (n = 4).

We first chose to determine the amount of nucleic acid and protein cargo extracted from Vn96-isolated EVs using the multiparametric extraction method. There is currently no consensus from commercial suppliers of EV isolation kits or the EV community for conditions used for pre-clearing of plasma samples^31–33^. We tested whether pre-clearing the plasma at 16,000× g would affect Vn96-DNA recovery. As shown in Fig. 3a, in 4 out of 9 donors (A-D) pre-clearing at 16,000× g caused a reduction in Vn96-DNA recovery, while in 5 out of 9 donors (E-I) pre-clearing had no effect. Based on these results we decided to omit a pre-clearing step. Figure 3b shows the amount of nucleic acid (DNA and RNA) and protein extracted from EVs isolated with the Vn96 synthetic peptide from 1 mL of plasma (n = 4). We also determined the percent coefficient of variance for the yield of DNA (16.5%) and RNA (20%) from 3 technical replicates in order to assess the precision of the method. These data demonstrate that by using Vn96 combined with our multiparametric extraction method we are able to reproducibly isolate appreciable amounts of DNA (10.8 ± 1.39 ng), RNA (16.8 ± 1.07 ng) and protein (209 ± 15.2 µg) from 1 mL of plasma (n = 4) that provide sufficient material for downstream analyses.

### Downstream analyses of EVs cargoes isolated by the multiparametric extraction method

**Figure 4:**
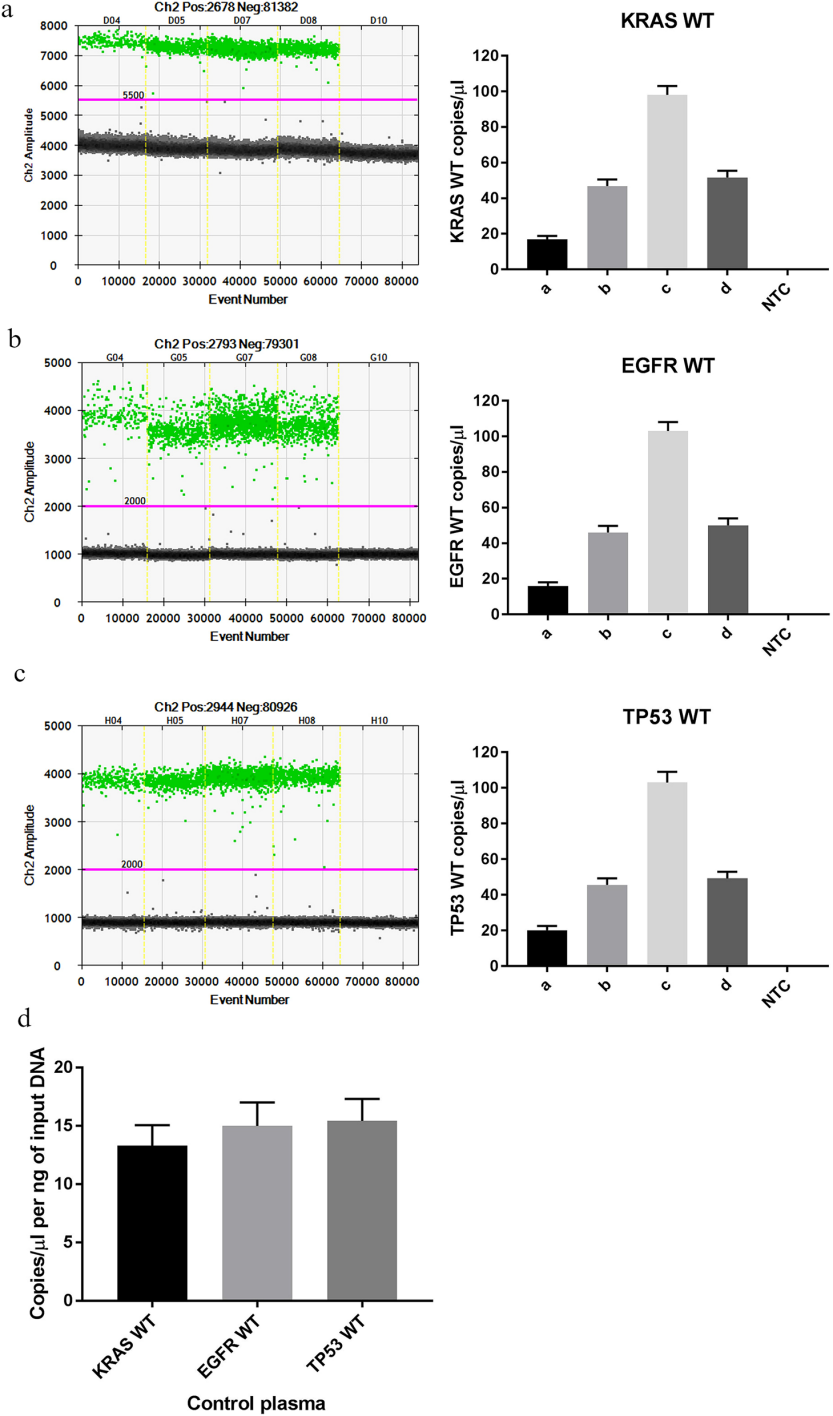
ddPCR analysis of DNA extracted from Vn96-isolated EVs from control patient plasma (n = 4). (**a**) *KRAS* WT ddPCR amplitude vs event number graph (left) and resulting *KRAS* WT copies ± Poisson confidence min and max for each control patient. Images were generated from BioRad QuantaSoft software (v1.7.4, https://www.bio-rad.com/en-ca/sku/1864011-quantasoft-software-regulatory-edition?ID=1864011, accessed on 28/01/2021). (**b**) *EGFR* WT ddPCR amplitude vs event number graph (left) and resulting *EGFR* WT copies ± Poisson confidence min and max for each control patient. (**c**) *TP53* WT ddPCR amplitude vs event number graph (left) and resulting *TP53* WT copies ± Poisson confidence min and max for each control patient. (**d**) Average normalized to DNA input copies ± SEM for *KRAS* WT, *EGFR* WT and *TP53* WT (n = 4).

DNA, RNA and protein extracted from Vn96-isolated EVs and cfDNA using the multiparametric extraction method were subsequently used for ddPCR detection for wildtype *KRAS*, *EGFR* and *TP53* genes, small RNA sequencing (sRNAseq), and mass spectrometry, respectively. Figure 4 shows the detection of *KRAS* WT (a), *EGFR* WT (b) and *TP53* WT (c). When normalized to the amount of DNA input (ng) for each ddPCR reaction the number of copies detected for *KRAS* WT, *EGFR* WT, and *TP53* WT are in concordance, indicating that plasma may contain unbiased genome coverage of DNA, as suggested previously^34^. These results demonstrate that the DNA (EV DNA and cfDNA) isolated via our multiparametric extraction method contains clinically-relevant material.

**Figure 5:**
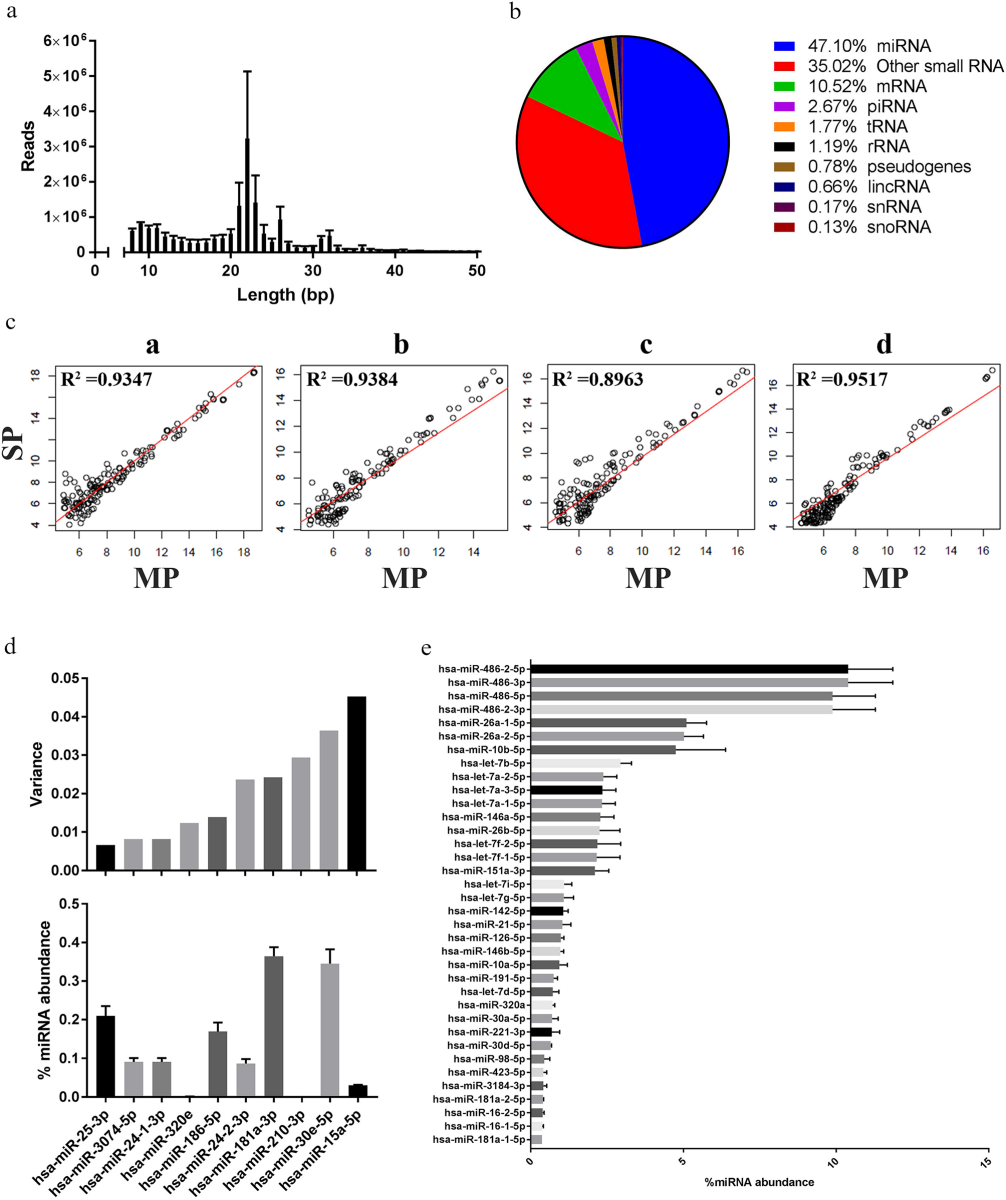
Small RNA sequencing (sRNAseq) results for RNA extracted from Vn96-isolated plasma EVs (n = 4). (**a**) Average number of reads ± SEM versus read length (bp). (**b**) Distribution of sRNA species in Vn96-isolated plasma EVs. (**c**) Regression plot of each patient miRNA result extracted by single parametric (SP) versus multiparametric (MP). (**d**) Top ten lowest variant miRNA (top) compared to their % of total miRNA abundance ± SEM (bottom). (**e**) Top 36 abundant miRNA expressed as % of total miRNA abundance ± SEM.

Numerous reports have used next-generation sequencing platforms to demonstrate the potential of EV RNA as a source of candidate biomarkers for the detection, prognosis and monitoring of treatment response for cancer^35–37^. As shown in Fig. 5, the RNA (n = 4) extracted from Vn96-isolated EVs via our multiparametric extraction method was used for sRNAseq. Total reads per sample were 16,419,954 ± 1,862,233, of which 12,452,808 ± 1,883,370 reads passed Ion torrent trimming and filtering. Figure 5a shows the average read lengths for the cDNA library generated from the EV RNA. A major peak between 21 and 23 bp and a minor peak between 31 and 32 bp were detected, which are typical for small RNA, such as miRNA. The percentage of mapped reads to reads passing filters was 78% ± 4% demonstrating that the RNA extracted from the Vn96-isolated EVs using our multiparametric method consists of *bona fide* RNA species. As shown in Fig. 5b, almost half of the mapped reads were miRNA (47.1%), followed by “other” small RNA species (non-intergenic lncRNA, antisense RNA, etc.; 35.02%), mRNA (10.52%), piRNA (2.67%), tRNA (1.77%), rRNA (1.19%), pseudogenes (0.78%), lincRNA (0.66%), snRNA (0.17%) and snoRNA (0.13%).

In order to test whether our multiparametric extraction method affects the isolation of specific RNA species we also performed sRNAseq on RNA extracted from Vn96-isolated EVs from the same donor plasma using a single-parametric extraction method, namely the miRVana miRNA isolation (total RNA) kit. No statistically significant differences were detected for the total reads, reads passing filters, percentage of mapped aligned reads, and the percentage species of small RNA between multiparametric (MP) and single-parametric (SP) extraction methods. Lastly, the abundance of each miRNA detected in MP versus SP was also not statistically different, as shown in the log_2_ of abundance scatter plots depicted in Fig. 5c. The average R^2^ was 0.9303 ± 0.0119 (n = 4). These data indicate that capturing DNA on the Norgen Biotek Plasma/Serum Cell-Free Circulating DNA column does not affect downstream small RNA sequencing, and that use of the multiparametric extraction method does not deplete the sample of any potential small RNA biomarkers.

In an attempt to identify potential miRNA normalizers for Vn96-isolated EV RNA we analyzed the variance of individual miRNAs among donors and identified ten miRNAs that had the least variation with respect to abundance amongst the four individual donors (Fig. 5d). Interestingly, we identified hsa-miR-25-3p, hsa-miR-3074-5p and hsa-miR-24-1-3p as having the lowest variance amongst the different donors as well as moderate abundance (Fig. 5d). Lastly, as shown in Fig. 5e, the vast majority of the aligned miRNA we identified in the EVs isolated from donor plasma belonged to the hsa-miR-486 family, which represented ~ 40% of the aligned miRNA reads. Some additional notable top 36 abundant miRNAs identified are those that have been suggested to be EV-specific^38^, including the hsa-miR-let-7a family and hsa-miR-146b-5p. In addition, we tested whether specific miRNA are protected in EVs by treating the Vn96-EV pellet with RNAse A prior to multiparametric extraction, following previously published protocols^39,40^. As shown in Supplementary Fig. 4, miR-16 and let-7b-5p are protected in EVs from RNAse A treatment.

**Figure 6:**
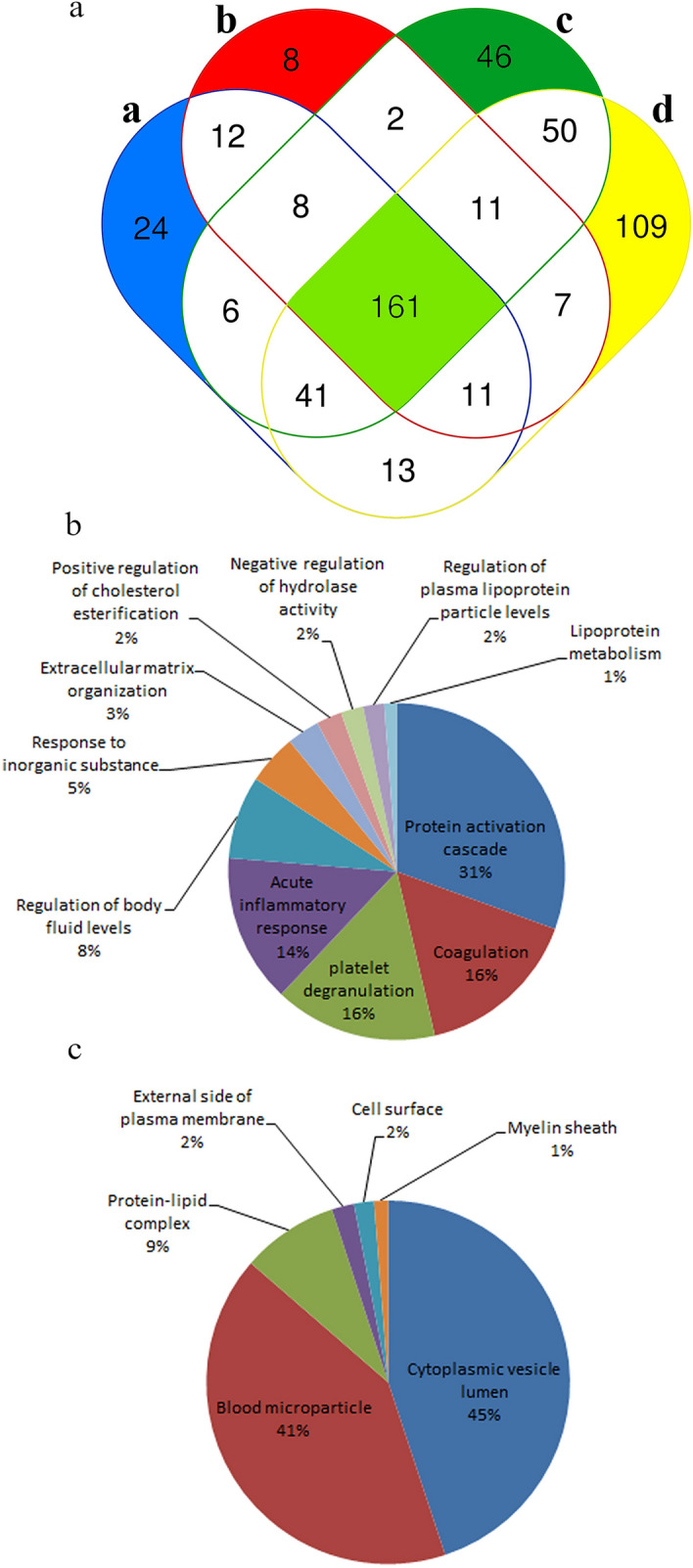
Mass spectrometry results for proteins extracted from Vn96-isolated plasma EVs (n = 4). (**a**) Venn diagram demonstrating the overlap of proteins from each patient. A web-based tool was used to generate Venn diagrams (http://bioinformatics.psb.ugent.be/webtools/Venn/, accessed on 28/01/2021). Gene ontology analysis categorized by biological process (**b**) and cell component (**c**). Revigo web-based tool was used to generate figures. ^24^

Mass spectrometry (MS) is one technique used to enable the discovery of new protein biomarkers and has recently been used as a platform to analyze Vn96-isolated EVs from cell culture media and urine^16,41,42^. Protein (25 ug) was extracted using our multiparametric protocol from Vn96-isolated EVs from 4 donor plasma samples and subjected to MS analysis; each plasma sample was analyzed in triplicate and the common set of proteins from the three MS experimental replicates was compiled for analysis (Fig. 6; Table S1). The average number of proteins identified by MS from all 4 Vn96-EV samples was 310 ± 43 (Donors: a = 276, b = 220, c = 325 and d = 421). As shown in Fig. 6a, we generated a 4-way Venn diagram in order to demonstrate the differences and commonalities among the 4 plasma-EV samples. A common core set of 161 proteins from a total of 348 unique proteins, representing ~ 46% commonality, was identified in the samples. Validation by Western blotting of two proteins identified by MS is shown in supplemental data (Figure S2). Interestingly, we detected a significant number of proteins that are exclusive to each sample, demonstrating the potential biological variability among individuals for a plasma EV protein analysis. As such, donors a through d had 24, 8, 46, and 109 unique Vn96-isolated EV proteins, respectively.

Next we analyzed the common core set of 161 proteins by gene ontology in order to classify the proteins into their biological processes (b) and cell component (c). Not surprisingly, the proteins identified were assigned biological processes most commonly found in blood, including protein activation cascade (31%), coagulation (16%), platelet degranulation (16%), and acute inflammatory response (14%), among others (Fig. 6b). In addition, the proteins identified fell mainly into two cell components, which were cytoplasmic vesicle lumen (45%) and blood microparticle (41%), thereby providing further evidence of the ability of Vn96 to isolate EVs (Fig. 6c). Lastly, we compared our common core set of proteins using PantherDB (http://geneontology.org, accessed on 28/01/2021) to identify cellular components and found that 112 out of our 161 proteins are associated with “Extracellular Exosome” (*p* = 4.54E^-75^).

Together, our analyses of DNA, RNA, and protein obtained from Vn96-isolated EVs and cfDNA using our multiparametric extraction method demonstrate that this material is amenable to downstream biomarker analysis using standard methods, such as ddPCR, small RNA sequencing, and mass spectrometry, and provides an opportunity to perform multi-omic analyses from the same limited sample.

## Discussion

The Vn96 synthetic peptide has been recognized as an excellent technology for EV isolation from biofluids because it does not require specialized equipment, is time efficient, and captures biomaterial (DNA, RNA and protein) that is amenable to biomarker analyses. Additionally, Vn96 is able to co-isolate cfDNA from biofluids^15^, providing additional value of this method over other EV isolation methods for circulating biomarker analyses. We now report the development of a streamlined and simplified workflow for Vn96 peptide-mediated isolation of EVs and cfDNA from plasma. We demonstrate that the Vn96 peptide efficiently isolates plasma EVs that have characteristic EV protein markers, such as CD63, CD9, Flotillin1, and HSC70. We also demonstrate that the size of EVs isolated with Vn96 is homogeneous in comparison to UCF and is similar to the EV size obtained by scUCF. Using this new workflow for Vn96-mediated EV and cfDNA isolation from plasma, we developed a multiparametric extraction protocol to sequentially extract DNA, RNA and protein from the same isolated material for subsequent clinically-relevant biomarker analysis (ddPCR) and multi-omic analyses (sRNAseq and MS). As biomarker analysis studies frequently employ the analysis of a single type of biomarker (DNA, RNA or protein), our multiparametic extraction protocol represents an advance for efficient biomarker analysis that conserves valuable patient samples, and provides a means to assay multiple types of biomarkers (DNA, RNA and protein) from the same starting material to enable a comprehensive analysis of molecular changes in a disease.

EVs have gained significant attention as a material for liquid biopsy tests^43,44^. Indeed, several EV-specific diagnostic biomarkers are now in development for a variety of diseases^45,46^. Despite the recognition that EVs contain all types of parameters for biomarker detection (e.g. DNA, RNA, protein), their use in diagnostic tests has been hindered by a lack of clinically-amenable methods to isolate EVs from biofluids, such as plasma. We have previously demonstrated that the protocol used to isolate EVs with the Vn96 synthetic peptide can be adapted for use in diagnostics labs with minimal requirements for specialized equipment or unacceptably long timeframes^14^. We have now optimized our method for Vn96-mediated EV isolation from patient plasma and show that Vn96 efficiently isolates a broad spectrum of EVs from plasma (Fig. 2), thereby providing a rich starting material for biomarker analyses.

A comprehensive analysis of biomarkers found in the multiple types of material harboured by EVs could provide improvements in the specificity and sensitivity of disease detection via biomarkers. For example, a DNA mutation analysis may not detect any therapeutically-relevant mutations in a cancer patient’s circulating tumour DNA (ctDNA), whereas such a patient may have a miRNA expression signature that indicates disease presence. An analysis of both the DNA and RNA contained in the same EV population could permit a comprehensive molecular analysis to detect disease markers that a single parametric approach may miss. An added feature of the Vn96 synthetic peptide is its ability to co-isolate both EVs and cfDNA from biofluids (^15^; Figure S1), such as plasma, providing a rich source for DNA mutation detection. With this in mind, we sought to develop a multiparametric extraction method for Vn96-isolated material that (1) conserves the amount of patient plasma used for diagnostic assays by making efficient use of the isolated material, (2) streamlines the time and effort required to obtain each type of analyte for biomarker analysis, (3) utilizes a protocol that can be adapted to a clinical diagnostics lab environment and (4) maximizes the amount of molecular ‘omic data from EVs and cfDNA to enable multi-omic profiling. Conservation of the amount of plasma utilized is an important consideration for biomarker discovery studies that make use of biobank samples, where only small amounts of plasma may be available for analysis. An efficient use of the isolated material is important because despite the efficiency of EV and cfDNA isolation using the Vn96 peptide, there is a finite amount of material available for downstream analyses. Separate workflows for the isolation of plasma DNA, RNA and protein increase the time and effort required to generate material for biomarker analysis and slows the pace of discovery and/or biomarker testing. Finally, to have an impact on patient care the method must be adaptable to a clinical diagnostic laboratory setting. Moreover, a growing trend in the field of biomarker discovery is the use of machine learning and artificial intelligence algorithms in order to better diagnose and/or predict complex diseases, such as cancer. This type of approach is enabled by a great number patient samples (n) and improved by the complexity of the data associated with each patient (e.g. multi-omic datasets).

Our multiparametric extraction protocol achieves the above-listed goals by (1) requiring only 1–4 mL of patient plasma, (2) permitting sequential extraction of DNA, RNA, and protein with minimal handling from the same EV and cfDNA isolation, (3) using methods that can be performed in a clinical diagnostics laboratory, and (4) providing material that is amenable to multi-omic analyses.

We believe the workflow we have developed for Vn96-isolated EV and cfDNA analyses is an important step toward the fulsome deployment of EV-based diagnostic and prognostic assays in a clinical lab setting. Future investigations using our EV and cfDNA isolation and extraction protocols will determine whether multiparametric biomarker analyses of EV cargoes can improve the sensitivity and specificity of biomarkers for disease detection.

## Supplementary Information

Supplementary Information 1.

Supplementary Information 2.
