# Supplementary material for: A multiparametric extraction method for Vn96-isolated plasma extracellular vesicles and cell-free DNA that enables multi-omic profiling
Source: Sci Rep. 2021 Apr 13;11:8085. doi: 10.1038/s41598-021-87526-y (PMC8044196; doi:10.1038/s41598-021-87526-y)
Supplement: Supplementary file 2 — Supplementary Information 2. [file 41598_2021_87526_MOESM2_ESM.docx]

A multiparametric extraction method for Vn96-isolated plasma extracellular vesicles and cell-free DNA that enables multi-omic profiling

Jeremy W. Roy^1^, Catherine A. Taylor^1^, Annie P. Beauregard^1,2^, Surendar R. Dhadi^1^, D. Craig Ayre^1,3^, Sheena Fry^1,4^, Simi Chacko^1^, Gabriel Wajnberg^1^, Andrew P. Joy^1^, Ngoc-Nu Mai-Thi^1^, Nicolas Crapoulet^1^, David A. Barnett^1^, Anirban Ghosh^1,5^, Stephen M. Lewis^1,5,6^, and Rodney J. Ouellette^1,5,7^

Author Affiliations:

^1^ Atlantic Cancer Research Institute, Moncton, New Brunswick, Canada

^2^ Current Address: Fisheries and Oceans Canada, Aquatic Animal Health, Moncton, New Brunswick, Canada

^3^ Current Address: Immunology, Genetics and Molecular Sciences, University of Medicine and Health Sciences, St. Kitts and Nevis

^4^ Current Address: Specialized Health Services Directorate, Health Canada, Ottawa, ON, Canada

^5^ Department of Chemistry & Biochemistry, Université de Moncton, Moncton, NB, Canada

^6^ Beatrice Hunter Cancer Research Institute, Halifax, NS, Canada

^7^Corresponding author:

Dr. Rodney Ouellette

35 Providence St., Moncton, NB, E1C 8X3 Canada

Phone: 1-506-862-7512

[rodneyo@canceratl.ca](mailto:rodneyo@canceratl.ca)

**
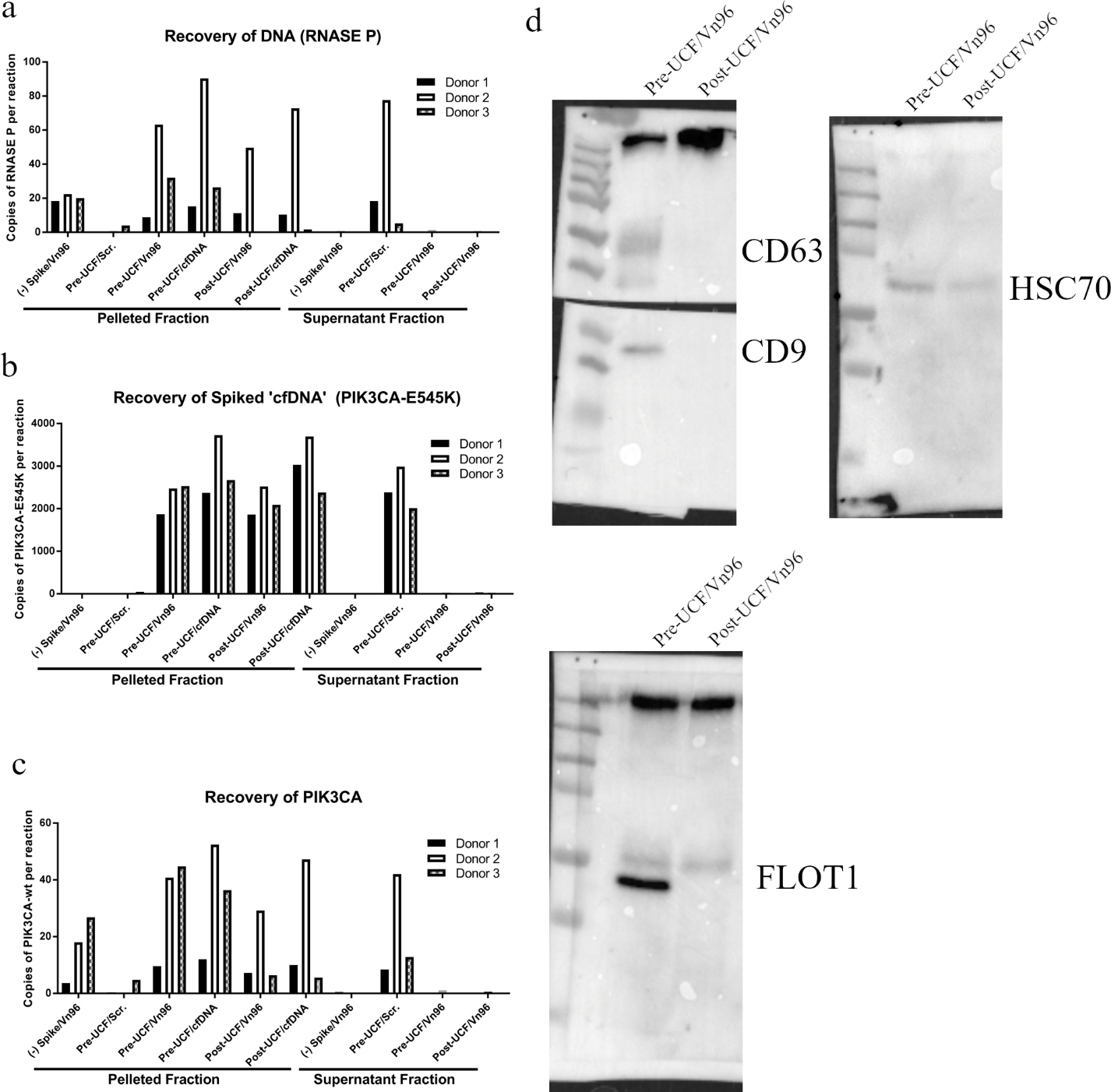
**

**Supplementary Figure 1**: Plasma from three healthy human donors was diluted 1:3 in sterile PBS. Half of the diluted plasma was subjected to ultracentrifugation (UCF) and the resulting supernatant (post-UCF) was retained as EV-depleted plasma. Both pre-UCF and post-UCF plasma was supplemented with 100 fg/mL of a 220 base pair gBlocks gene fragment corresponding to PIK3CA-E545K and material was isolated from the supplemented plasma using Vn96. A scrambled peptide (Scr.) was included as a negative control for non-specific DNA isolation. DNA was extracted from the Vn96 or scrambled peptide pellets (pelleted fraction) or directly from the plasma supernatant after Vn96 incubation (supernatant fraction) using the Qiagen Circulating Nucleic Acid Kit (Qiagen, cat # 55114). Cell-free DNA (cfDNA) isolation directly from supplemented plasma was included as a positive control. a) Recovery of total DNA was quantified by ddPCR using an RNASE P TaqMan Assay. b) PIK3CA (wild-type) and c) supplemented PIK3CA-E545K gBlock DNA fragments were quantified using a PIK3CA-E545K TaqMan Assay by ddPCR (Thermo Fisher cat# Hs000000086_rm). d) EVs were recovered from either pre- or post-UCF plasma using Vn96 and the pelleted material was analyzed by Western blot using antibodies against canonical EV markers in order to verify removal of EVs from plasma by UCF. For CD63 and CD9 blot was cut prior to hybridization with respective antibodies.

PIK3CA-E545K gBlock DNA Fragment (Integrated DNA Technologies) sequence (location of single nucleotide polymorphism in bold): attattttat tttacagagt aacagactag ctagagacaa tgaattaagg gaaaatgaca aagaacagct caaagcaatt tctacacgag atcctctctc tgaaatcact **a**agcaggaga aagattttct atggagtcac aggtaagtgc taaaatggag attctctgtt tctttttctt tattacagaa aaaataactg aatttggctg atctcagcat

**
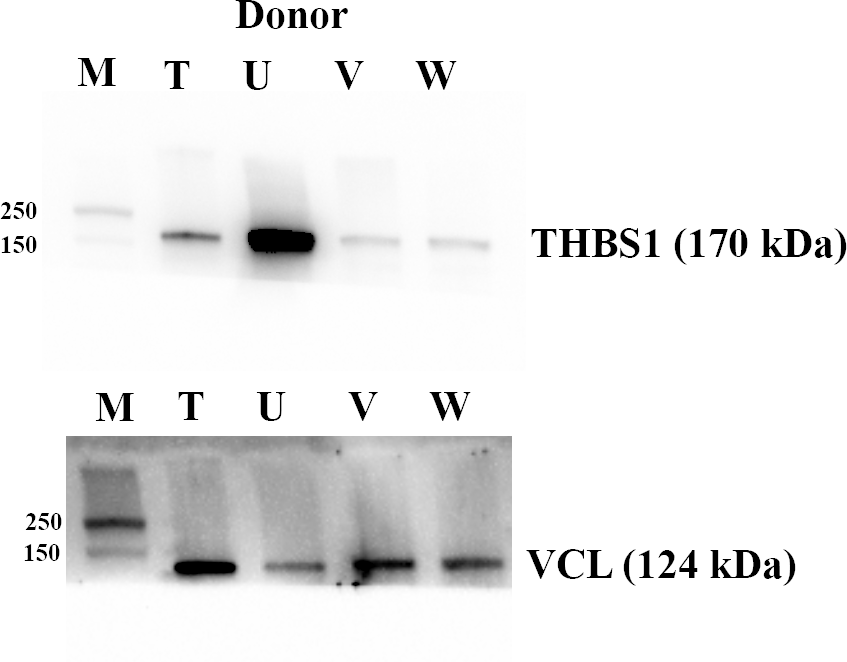
**

**Supplementary Figure 2.** Validation of mass spectrometry data by Western Blot. M = molecular weight Marker. Western blot was performed as described in Materials and Methods. THBS1 and VCL antibodies were purchased from Cell Signalling Technologies and used at 1:1,000 dilution. Blots were cut prior to hybridization with antibodies.


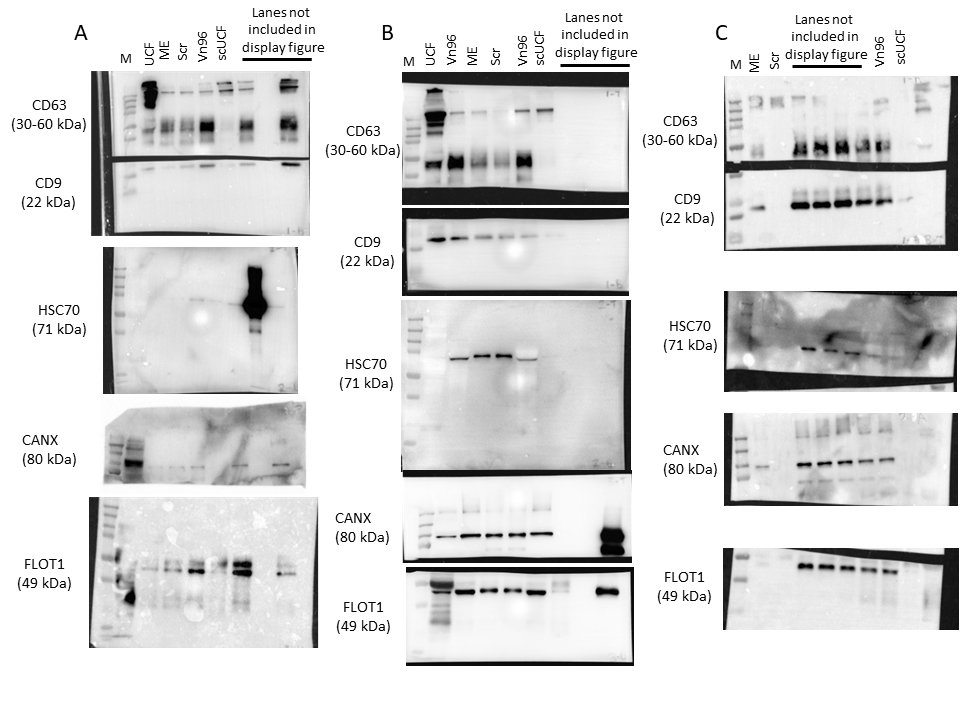


**Supplementary Figure 3**. Full-length Western blot images of all biological replicates for display Figure 2A. EVs were captured from donor plasma (n = 3) with UCF, ME buffer (ME), scrambled peptide (Scr), Vn96 (100 µg), and sucrose-cushion UCF (scUCF) and protein was isolated. Western blot was performed and probed for CD63 and CD9, HSC70, CANX and FLOT1. Panels A, B and C represent biological replicates and panel A is full length blots for the manuscript display figure. Protein loading marker (M). In some cases membranes were cut and the resulting portions subjected to hybridization with various antibodies (e.g. CD63 and CD9; CANX and FLOT1).

**Supplementary Figure 4.** ddPCR results for normalized to exogenous spike-in miRTC miR 16 (a) and let 7b-5p (b) for Vn96-EV pellets treated with or without (w/o) RNAse A (30 µg/ml) for 15 min (Enderle D. *et al*. 2015 and Huang X. *et al*. 2013). cDNA was generated using miScript II RT kit (Qiagen) and ddPCR was performed on a BioRad QX200 digital PCR system with miScript primer assays (miRTC: Assay id MS0000001; miR-16: Assay id MS00031493; let 7b-5p: Assay id MS00003122) and universal reverse primer similar to our previous publication (Saucier D. *et al*. 2019). ns = not statistically significant.

**Supplementary Table 1**. Proteomic data for each donor sample. The number of peptides, protein spectral matches (PSMs) and the protein group gene name.
